# Evaluating the need for magnesium administration following cardioplegic arrest with del Nido cardioplegia solution

**DOI:** 10.1051/ject/2024033

**Published:** 2025-03-07

**Authors:** Carrie Whittaker Striker, James A. Reagor

**Affiliations:** 1 Department of Cardiovascular Perfusion, Children’s Wisconsin Milwaukee Wisconsin USA; 2 Department of Cardiovascular Perfusion, Cincinnati Children’s Hospital Medical Center and Department of Pediatrics, University of Cincinnati College of Medicine Cincinnati Ohio USA

**Keywords:** del Nido, Cardioplegia, Magnesium, Hypomagnesemia, Arrhythmia

## Abstract

*Background*: del Nido cardioplegia (dNC) solution is widely used in pediatric and congenital cardiac surgery. In 2014, Cincinnati Children’s Hospital Medical Center (CCHMC) changed from Mee cardioplegic solution to dNC. Since Mee solution does not contain magnesium, magnesium was administrated post cross-clamp removal, at a dose of 25 mg/kg up to 1 g, to abate hypomagnesemia. This practice remained in place with the use of dNC. We postulated that patients may experience hypermagnesemia under this protocol. *Methods*: To determine if exogenous magnesium is necessary post-clamp removal in our practice, a study examining serum magnesium levels during cardiopulmonary bypass (CPB) was completed from January 2022 through October 2023 (IRB #2021-0816). One hundred patients undergoing CPB with cross-clamp, ranging from infants to adults, were consented. Two magnesium samples were collected. Draw 1 (D1) was collected post cardioplegia administration and 30 min prior to cross-clamp removal. Draw 2 (D2) was collected post-cross-clamp removal and 10 ± 2 min following magnesium administration. *Results*: Both samples demonstrated magnesium levels > 1.6 mg/dL or higher (normal magnesium range at CCHMC, 1.6–2.6 mg/dL). A Wilcoxon rank sum test demonstrated statistical significance for D1, comparing the number of samples that fell above 2.6 mg/dL vs. those that fell within the normal range (*p* < 0.001). D2 demonstrated values above the normal range for all but one sample, which does not satisfy the criteria of the Wilcoxon rank sum test for demonstrating significance (*p* = 0.089); however, ninety-nine samples displayed hypermagnesemia. Conclusion: This study demonstrates that exogenous magnesium administration may not be necessary in the setting of our practice at CCHMC and dNC cardioplegic arrest.

## Introduction

The cardioplegia solution for many pediatric and congenital cardiac surgical programs is del Nido Cardioplegia (dNC). Compared to other cardioplegic solutions, dNC accommodates longer ischemic times between dosing allowing the surgeon increased intervals of uninterrupted operating time [1]. At Cincinnati Children’s Hospital Medical Center (CCHMC), dNC has been used since 2014, replacing Mee solution. Unlike dNC (Table 1), Mee solution (Table 2) does not contain magnesium hence, the institutional practice was to administer magnesium after cross-clamp removal at a dose of 25 mg/kg to a maximum dose of 1 g.

**Table 1:** del Nido cardioplegia solution.

Constituents	Quantity
Plasma-Lyte A	1 L
Potassium (KCL)	26 mEq
Magnesium (MgSO_4_)	2 gm
Sodium Bicarbonate (NaHCO_3_)	12 mEq
Mannitol	3.26 gm
Lidocaine	130 mg
Total volume	1059 mL

**Table 2:** Mee cardioplegia solution.

Constituents patients < 10 Kg	Quantity
Sterile water	353 mL
Albumin 25%	100 mL
Potassium (KCL)	15 mEq
Sodium bicarbonate (NaHCO_3_)	26 mEq
Mannitol 20%	2.5 gm
Calcium chloride (CaCl_2_)	52 mg
Dextrose 50%	5.63 mL
NaCl	23.28 mEq
Total volume	511 mL

Constituents patients > 10 Kg	Quantity

Sterile Water	706 mL
NaCl 0.9%	200 mL
Potassium (K^+^)	30 mEq
Sodium Bicarbonate (NaHCO_3_)	10 mEq
Mannitol 20%	10 gm
Calcium Chloride (CaCl_2_)	104 mg
Dextrose 50%	11.26 mL
NaCl	46.56 mEq
Total Volume	1005 mL

Since the transition to dNC cardioplegia, the administration of magnesium post-cross-clamp removal remained in place. To continuously interrogate our clinical processes, specifically following major practice changes, a study was designed to evaluate serum magnesium levels on CPB. We hypothesized that magnesium levels, under CCHMC’s current protocol, may result in magnesium levels at or above physiologic levels. This study examined magnesium levels prior to and after cross-clamp removal for one hundred congenital cardiac patients undergoing cardioplegic arrest with dNC and magnesium administration from January 2022 through October 2023 at CCHMC.

## Materials and methods

“Magnesium Administration After Cardioplegic Arrest with Del Nido Cardioplegia” (IRB # 2021-0816) was approved in January 2022 by the CCHMC institutional review board. This study measured serum magnesium levels pre and post-cross-clamp removal for patients undergoing cardiopulmonary bypass (CPB) in dNC arrest and magnesium administration. Patients were recruited based on the cardiac surgical schedule and appropriate informed consent was obtained. Exclusions for participation included patients who did not receive dNC or those who did not receive magnesium sulfate administration of 25 mg/kg up to 1 g post-cross-clamp removal.

### Conduct of CPB

Cardiopulmonary bypass was conducted in a similar fashion by eight pediatric and congenital perfusionists, in adherence with department guidelines, for four congenital cardiothoracic surgeons. Age and weight-appropriate circuitry and a 3.0 cardiac index flow target based on body surface area were utilized. Bypass circuits were comprised of oxygenators from the Terumo FX series (Terumo Cardiovascular, Ann Arbor, MI, USA), tubing coated with Terumo’s X-Coating (Terumo Cardiovascular, Ann Arbor, MI, USA), and either the DHF02 or DHF06 hemoconcentrator (LivaNova, London, UK). Roller pumps were used with appropriately sized pump boots for all cases and roller pump occlusion was set for each case by the pressure drop method (~2 mmHg drop in pressure per second). CPB circuits were primed with packed red blood cells (PRBC) as necessary to ensure a post-dilutional hematocrit greater than 25% on bypass. PRBC units were processed in a Fresenius Kabi Continuous Autotransfusion System (CATS) cell savage device (Terumo Cardiovascular, Ann Arbor, MI, USA) and washed with Plasmalyte-A (Baxter Healthcare, Deerfield, IL, USA) before administration into the CPB pump. Patients weighing less than eight kilograms received at least one full unit of fresh frozen plasma (FFP) preserved in Anticoagulant Citrate Dextrose (ACD-A) solution (Baxter Healthcare, Deerfield, IL, USA), 120 mLs of which was administered via the CPB prime upon initiation of bypass and the rest of the unit given during CPB. In the event, that patients received two or more units of PRBCs during CPB, one unit of FFP for every two units of PRBC was administered during CPB. No platelets or cryoprecipitate were administered during CPB. Transfusion of PRBC during CPB and/or the use of modified ultrafiltration post-CPB was indicated to maintain an on-bypass hematocrit above 25% and to ensure a hematocrit greater than 30% for post-repair acyanotic patients and greater than 40% for cyanotic patients. Bypass for patients with cyanotic lesions, patients with a pO_2_ less than 80 mmHg, was initiated at a cyanotic pO_2_ of approximately 50 mmHg. The pO_2_ upon initiation of CPB for acyanotic patients was approximately 200 mmHg. Bypass was initiated upon reaching a target heparin concentration as determined by individual heparin dose response (HDR), targeting an activating clotting time (ACT) of 480 s and minimum ACT of 400 s measured via the Medtronic Heparin Management System (HMS), (Medtronic, Minneapolis, MN, USA). An ACT of greater than 480 s and a heparin concentration greater than or equal to the HDR target were maintained during CPB and measured every 30 min. Depending upon the extent of the repair necessary, patients were cooled as low as 28 °C, measured via bladder temperature, and rewarmed to 36 °C prior to termination from bypass. Conventional ultrafiltration was utilized during CPB with a target fluid balance of 0 to −40 mL/kg during the period of CPB and MUF [2]. Dilutional ultrafiltration (DUF) was performed with Plasma-Lyte A (Baxter Healthcare, Deerfield, IL, USA) with 20 mEq sodium bicarbonate and 200 mg calcium chloride per liter. DUF clearance volumes targeted 50–200 mL/kg during CPB. Upon stable termination of CPB, heparin was reversed based upon the heparin-protamine concentration titration measured within 30 min of CPB termination via the HMS. A post-protamine heparin concentration of zero and an ACT at or near baseline were considered to be acceptable heparin reversal criteria.

### Cardioplegia circuit setup and dosing regimen

Cardioplegia was administered via a nonrecirculating, custom circuit comprised of SMART-coated tubing and a CSC-14 heat exchanger (LivaNova, London, UK) containing approximately 100 mL of volume. dNC (Central Admixture Pharmacy Solutions, Cleveland, OH, USA) was administered upon cross-clamp application at a 20 mL/kg induction dose in a 1:4 ratio of blood to crystalloid. Maintenance doses of 10–20 mL/kg were administered as necessary at a frequency of every 90–120 min of cross-clamp time. Cardioplegia was administered at temperatures between 4 and 6 °C.

### Study procedure

Consented patients undergoing CPB with dNC arrest had two blood samples collected from the heart-lung machine sampling manifold and measured for magnesium levels. The first sample, Draw 1 (D1), was collected after cardioplegia administration and 30 min prior to cross-clamp removal. The second sample, Draw 2 (D2), was collected after cross-clamp removal and 10 ± 2 min after magnesium administration. Each sample, approximately 2 mL, was collected in a light green top tube and sent to the core lab at CCHMC for analysis. Table 3 illustrates the schedule of procedures for this study.

**Table 3:** Study sample schedule.

Magnesium sample	Sample time
1 mL blood removed via CPB circuit	30 min prior to cross-clamp removal and MgSO_4_ dose (Draw 1)
1 mL blood removed via CPB circuit	10 ± 2 min after cross-clamp removal and MgSO_4_ dose (Draw 2)

### Data collection

Magnesium levels were recorded on study-specific Case Report Forms (CRFs) and recorded in a RedCap (Vanderbilt University, Nashville, TN) database developed and maintained for the purpose of this study. Cross-clamp and magnesium administration times were collected during the normal clinical routine for patients undergoing congenital cardiac surgery with CPB. These data were electronically transmitted to the VISION (Spectrum Medical, Fort Mill, SC, USA) database. Data was secured in the password-protected RedCap and VISION database located within the CCHMC network during the study. The specific data contained in the VISION database was electronically harvested and submitted to the data management team for cleaning and correlation with data in the RedCap database.

### Measures

Magnesium levels were analyzed and compared against normally expected values. The normal expected value of blood serum magnesium is 1.60–2.60 mg/dL, per CCHMC lab reference values.

### Statistical analysis

The magnesium levels and cross-clamp times were described using medians and interquartile range (IQR). A Wilcoxon rank sum test was used to assess the difference in the magnesium levels between the magnesium ranges before and after magnesium administration, respectively. A paired t-test was used to estimate the difference in magnesium levels before and after the magnesium administration. Two-sided *p*-values < 0.05 were considered statistically significant. All statistical analyses were performed using R version 4.3.1 statistical software (R Core Team, 2020, Vienna, Austria).

## Results

**Figure 1:**
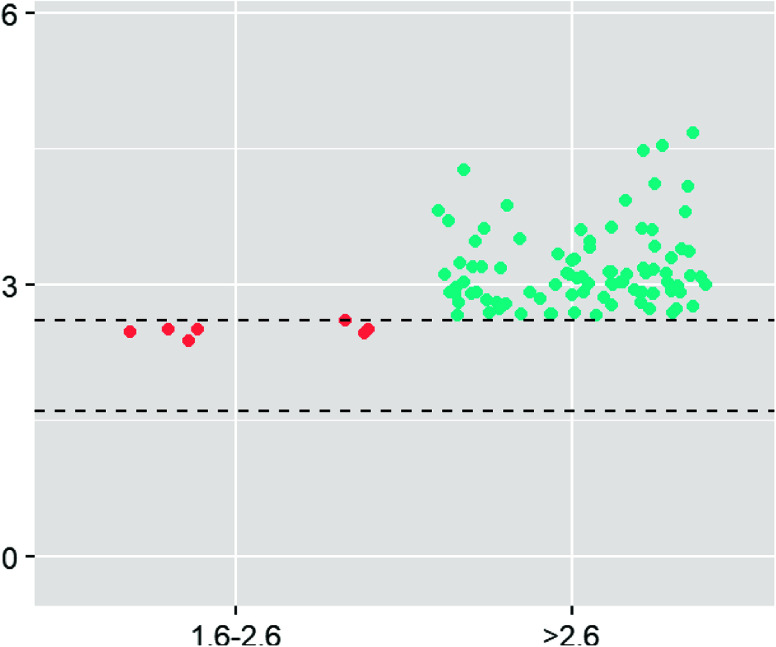
Draw 1 magnesium values grouped by those falling in the normal range of 1.6–2.6 mg/dL and greater than 2.6 mg/dL.

**Figure 2:**
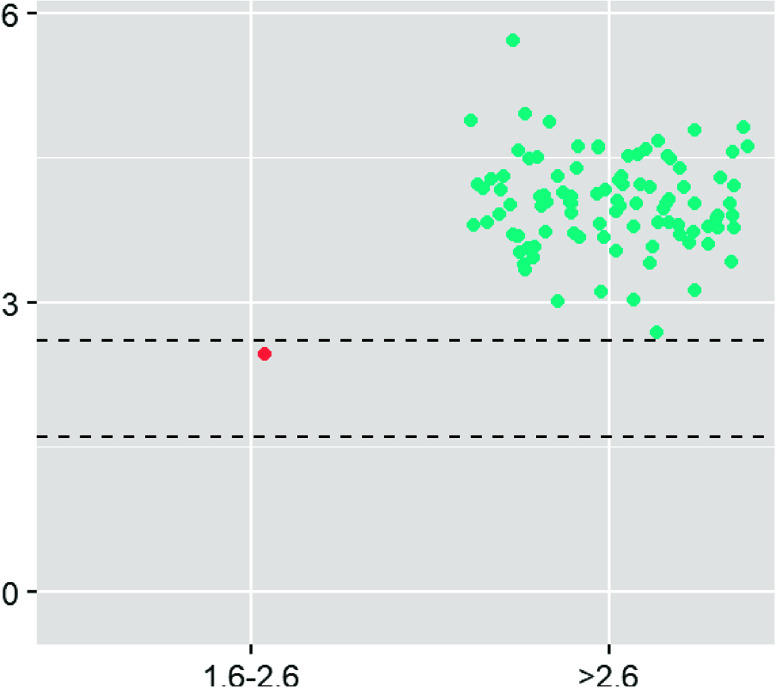
Draw 2 magnesium values grouped by those falling in the normal range of 1.6–2.6 mg/dL and greater than 2.6 mg/dL.

**Figure 3:**
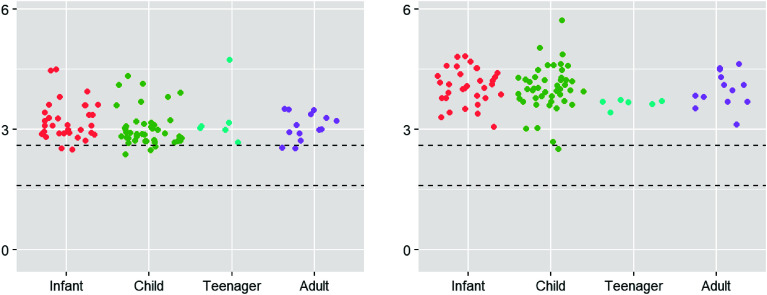
Magnesium levels within age brackets. Magnesium levels for infant (30 days to 1 year of age), child (1–12 years of age), teenager (13–17 years of age), and adult (18 or more years of age) subjects with Draw 1 magnesium levels in the left figure and Draw 2 on the right.

Magnesium levels for D1, prior to cross-clamp removal, and D2, post magnesium administration are shown in Figures 1 and 2. No magnesium levels fell below 1.6 mg/dL, the low end of normal. Median and mean values for D1 were 3.0 and 3.22 respectively, well above the top end of the 2.6 mg/dL normal range. A Wilcoxon rank sum test demonstrated statistical significance for D1 comparing the number of samples that fell above 2.6 mg/dL vs. those that fell within the normal range (*p* < 0.001). The median and mean values for D2 were 4.0 and 4.8 mg/dL respectively, with only one value falling in the normal range. D2 demonstrated values above the normal range for all but one sample, which does not satisfy the criteria of the Wilcoxon rank sum test for demonstrating significance (*p* = 0.089); however, ninety-nine samples displayed hypermagnesemia. The effect of magnesium administration was significant (*p* < 0.001), raising magnesium levels well above normal in almost all cases. When the magnesium levels were broken into age groups, similar trends continued with higher-than-normal magnesium levels at D1 and again at D2 (Figure 3).

## Discussion

Cardioplegic solutions are utilized to ameliorate metabolic injury of the myocardium during ischemia while providing a still, bloodless operative field. The efficacy of these solutions is demonstrated in the quiescence of cardiac activity and the propensity for post-ischemic recovery of acceptable ventricular function. Over time, cardioplegic solutions have been developed to improve recovery of the myocardium by attenuating myocardial reperfusion injury.

A major advancement in pediatric cardiac surgery was the introduction of del Nido cardioplegia. dNC was developed at the University of Pittsburgh during the 1990’s to address the needs of immature myocardium in neonates, infants, and pediatric patients [1, 3]. A characteristic of immature myocardium is a higher sensitivity to intracellular calcium related to an underdeveloped sarcoplasmic reticulum [1, 3]. In its formulation, dNC seeks to decrease intracellular calcium by including magnesium, a calcium-competing ion, and polarizing agents such as lidocaine [1]. Secondarily, dNC safely accommodates longer myocardial ischemic times thereby aiding surgical efficiency [1, 3–5].

dNC is a Plasmalyte solution (Baxter Healthcare, Deerfield, IL) with the following additives: mannitol, magnesium, sodium bicarbonate, potassium chloride, and lidocaine as listed in Table 1. These components were utilized with the following rationale. Mannitol, a free radical scavenger, exhibits osmotic properties which may reduce cardiac myocyte edema. Magnesium, a calcium channel blocker, attenuates the buildup of intracellular calcium in an effort to reduce diastolic stiffness post-cross-clamp removal, which may result in poor filling and decreased cardiac function [1, 6]. Sodium bicarbonate helps to maintain an adequate intracellular pH during anaerobic metabolism. Potassium chloride is utilized to establish a rapid depolarizing arrest by inhibiting the movement of sodium and potassium across the cellular membrane. Lidocaine assists in the blockade of the sodium channels and stabilizes the cellular membrane preventing intracellular movement of calcium and sodium, specifically during hyperkalemic arrest. dNC is delivered in a 1:4 ratio of blood to crystalloid (1).

Although dNC is most widely used in pediatric cardiac surgery and was initially developed for the immature myocardium, it has gained attention in adult cardiac surgery. Shu, Hong, Shen et al., demonstrate shorter ICU length of stay, less ventricular arrhythmia, increased hemodynamic stability resulting in earlier extubation, and decreased sternal wound infection (possibly due to less glucose) with del Nido over St. Thomas Solution [7]. Cayir and Yuksel demonstrated that the requirement for intraoperative defibrillation post cross-clamp removal was significantly less for adult cardiac CPB patients with the use of dNC over St. Thomas solution [5]. These findings are consistent with Buel, Striker, and O’Brien’s study in the congenital pediatric population [4]. Additionally, surgical efficiency is improved due to less dosing frequency of dNC over St. Thomas Solutions [5].

Magnesium is often administered following the release of the aortic cross-clamp for the prevention of dysrhythmias and ventricular dysfunction due to hypomagnesemia [8–10]. Hypomagnesemia post-CPB can be exacerbated by diuresis, hemodilution, and ultrafiltration during CPB. Previous studies have demonstrated that the administration of magnesium prior to cessation of CPB results in fewer arrhythmias and improved ventricular function [9–11]. These studies, however, did not use a magnesium-based cardioplegic solution such as dNC. As such, it is essential to determine if exogenous magnesium administration post aortic cross-clamp removal is necessary as hypermagnesemia can result in undesired effects such as arrhythmia and/or hypotension [12].

Continual evaluation of our clinical practices, specifically following an alteration in cardioplegic solutions, and considering the adverse effects of hypermagnesemia, it was prudent to ascertain magnesium levels on CPB. Under our current protocol, we hypothesized that the administration of exogenous magnesium post cross-clamp removal, in the setting of cardioplegic arrest with dNC solution, may result in hypermagnesemia. These data validate this hypothesis specifically with the use of a buffered Plasmalyte prime and dilutional ultrafiltration solution. Analysis of one hundred patients, regardless of age, exhibited serum magnesium levels at or above physiologic normal (1.6–2.6 mg/dL). Samples drawn prior to cross-clamp removal, D1 (*p* < 0.001), and post cross-clamp removal following magnesium administration, D2 (*p* = 0.089) demonstrate hypermagnesemia. Due to the deleterious effects of hypermagnesemia, it may be reasonable to eliminate routine administration of magnesium post-cross-clamp removal in the setting of dNC solution for cardioplegic arrest.

## Data Availability

The data supporting the findings of this research are not publicly available.
